# Neural Networks for Survival Prediction in Medicine Using Prognostic Factors: A Review and Critical Appraisal

**DOI:** 10.1155/2022/1176060

**Published:** 2022-09-30

**Authors:** Georgios Kantidakis, Audinga-Dea Hazewinkel, Marta Fiocco

**Affiliations:** ^1^Mathematical Institute Leiden University, Niels Bohrweg 1, 2333 Leiden, ZA, Netherlands; ^2^Department of Biomedical Data Sciences, Section Medical Statistics, Leiden University Medical Center (LUMC), Albinusdreef 2, 2333 Leiden, ZA, Netherlands; ^3^Department of Statistics, European Organisation for Research and Treatment of Cancer (EORTC) Headquarters, Ave E. Mounier 83/11, 1200 Brussels, Belgium; ^4^Population Health Sciences, Bristol Medical School, University of Bristol, Oakfield Grove, Clifton, Bristol BS8 2BN, UK; ^5^MRC Integrative Epidemiology Unit, Bristol Medical School, University of Bristol, Oakfield Grove, Bristol BS8 2BN, UK; ^6^Trial and Data Center, Princess Máxima Center for pediatric oncology (PMC), Heidelberglaan 25, 3584 Utrecht, UT, Netherlands

## Abstract

Survival analysis deals with the expected duration of time until one or more events of interest occur. Time to the event of interest may be unobserved, a phenomenon commonly known as right censoring, which renders the analysis of these data challenging. Over the years, machine learning algorithms have been developed and adapted to right-censored data. Neural networks have been repeatedly employed to build clinical prediction models in healthcare with a focus on cancer and cardiology. We present the first ever attempt at a large-scale review of survival neural networks (SNNs) with prognostic factors for clinical prediction in medicine. This work provides a comprehensive understanding of the literature (24 studies from 1990 to August 2021, global search in PubMed). Relevant manuscripts are classified as methodological/technical (novel methodology or new theoretical model; 13 studies) or applications (11 studies). We investigate how researchers have used neural networks to fit survival data for prediction. There are two methodological trends: either time is added as part of the input features and a single output node is specified, or multiple output nodes are defined for each time interval. A critical appraisal of model aspects that should be designed and reported more carefully is performed. We identify key characteristics of prediction models (i.e., number of patients/predictors, evaluation measures, calibration), and compare ANN's predictive performance to the Cox proportional hazards model. The median sample size is 920 patients, and the median number of predictors is 7. Major findings include poor reporting (e.g., regarding missing data, hyperparameters) as well as inaccurate model development/validation. Calibration is neglected in more than half of the studies. Cox models are not developed to their full potential and claims for the performance of SNNs are exaggerated. Light is shed on the current state of art of SNNs in medicine with prognostic factors. Recommendations are made for the reporting of clinical prediction models. Limitations are discussed, and future directions are proposed for researchers who seek to develop existing methodology.

## 1. Introduction

There is a growing interest by the medical community in applying machine learning (ML) to predict clinical outcomes [1]. This interest springs from the collection of large-volume patient information in electronic health records, and the growing availability of mixed data, for instance clinical and molecular. ML techniques are assumption-free and data-adaptive, making them attractive for modelling complex data. Artificial neural networks (ANNs) and other ML techniques have been used in healthcare for clinical diagnosis, prediction, and to support decision making, e.g., in the domains of cancer and cardiology [2, 3].

Survival analysis (also called time-to-event analysis) is used to estimate the lifespan of a particular population under study. Survival data are omnipresent in medicine where the focus is on modelling a particular event of interest (for example disease-progression or death). This kind of data are often right-censored; they can be seen as a specific type of missing data in which time to the event of interest may be unobserved, either due to subjects being lost to follow-up, or due to time limitations such as study termination. The presence of censored observations makes the analysis of these data and the direct application of ML algorithms challenging, requiring modifications to the traditional approaches. As such, prediction of survival outcomes with ANNs, one of the most popular machine learning techniques in healthcare—poses unique hurdles with respect to the development and use of effective algorithms that can deal with right censoring (main focus here).

The most popular statistical model to analyse time-to-event data in medical research is the Cox proportional hazards defined as *λ*(*t*|*X*) = *λ*_0_(*t*)exp(*X*^*T*^*β*), where *X* is the vector of covariates, and *λ*_0_(*t*) is the baseline hazard function which is left unspecified. The effect of the covariates on the hazard is modeled by the parametric part exp(*X*^*T*^*β*) leading to the proportional hazard regression model [4]. Possible alternatives include parametric regression methods which make strong assumptions about the time distribution (e.g., exponential, Weibull, or log-normal) and flexible nonparametric methods that do not make any prior assumptions regarding the time or the predictors (e.g., Random Survival Forest and ANNs) [5–7]. A well-known nonparametric method to estimate the survival function was proposed by Kaplan-Meier [8]. It is used to estimate the fraction of patients alive after a specific starting point (for example, start of treatment).

ANNs have been widely used for survival data. Two decades ago, Ripley B. and Ripley R. published an overview that identifies the most appropriate survival neural networks (SNNs) for medical applications [9]. In their paper, they show different ways of adapting classification networks to survival data and describe the disadvantages of these methods. An example of a work outside the medical field is discussed by Baesens et al. [10]; in this work, various SNNs in context of personal loan data are used where the performance is compared to the Cox proportional hazards model [4]. In a recent comprehensive survey, Wang et al. [11] discuss conventional and modern methods for survival analysis with right-censored data. The authors conclude that SNNs are well-suited to predicting survival and estimating disease risk and are able to provide personalised treatment recommendations. Nevertheless, despite their nonnegligible development in medicine for time-to-event data, a comprehensive review on SNNs using prognostic factors is missing. Prognostic factors are patient/disease characteristics (such as age, sex, or disease stage), which can be used to estimate the impact on survival, disease recurrence, or on others clinical outcomes. Typically prognostic factors do not include images (pathology images, tumor slices, whole slide images, etc.) or genetic marker sequences of DNA (variables from the area of bioinformatics).

In this article, we fill this gap with a structured overview of SNNs in clinical prediction with prognostic factors which can be used as a guideline for future research. We aimed at providing a broad understanding of the literature (1st January 1990-31st August 2021), as part of a growing trend towards personalised medicine [1]. We discuss how SNNs are employed in the medical field for prediction and detail how researchers have tried to adapt a classification method to right-censored survival data. During the 1990s, there were several modelling attempts, followed by a stagnation in scientific publications. In the past years, however, the advancement of machine learning has led to an increased interest from the medical community, where neural networks are now viewed as a promising modern approach to modelling medical data. In this review, we distinguish, following a chronological order, between methodological manuscripts (novel method or a new theoretical model) or applications that may build on existing methods to improve or adapt them based on the data at hand. The major distinctions between SNNs are 3-fold: (a) data structure: some authors rely on a long format transformation of the dataset, whereas others use the original dataset; (b) incorporating time information in the SNN: time is either added as part of the input features of the SNN, while specifying a single output node, or this step is omitted and multiple output nodes are specified—one for each time interval; (c) estimation of outcome (output layer of the networks): some SNNs predict survival probabilities directly, while others estimate (conditional) death probabilities (hazard), from which the former can be calculated.

This work is supplemented with a critical appraisal on model aspects to be designed and reported more carefully in future studies. Key characteristics of prediction models (i.e., number of patients/predictors, evaluation measures, validation, and calibration) are listed for methodological papers and applications, and the predictive performance of SNNs is compared to the Cox proportional hazards model (if reported in the papers). We conclude with recommendations on the correct application of SNNs in context of clinical prediction models and discuss limitations and potential directions of future research. Particular interest is on SNNs applied to cancer prediction in contrast to other medical fields.

This manuscript is organised as follows: in section “sec: conducting-the-review”, we describe our search and review strategy. Section “sec:methodologies” focuses on the various SNN approaches identified. We present in a chronological order: “sec:methodologies-early-methodological-approaches”, “sec:methodologies-approaches-new-millenium”, and “sec:methodologies-modern-methodological-approaches”. Section “sec:applications” summarizes 11 applications to real or simulated data. In section “sec:a-critical-perspective”, we perform a critical appraisal of relevant studies, considering their “subsec:general_study_characteristics”, “subsec:model_development” aspects, “subsec:model_validation”, and “subsec:comparison_with_Cox”. Section “sec:discussion” provides a discussion of current limitations and future directions.

## 2. Conducting the Review

We searched the Medline biomedical database from 1st January 1990 to 31st August 2021, and identified 261 relevant studies where survival prediction was estimated using ML techniques. An additional 15 studies were identified by looking at references of selected papers and a previous literature overview by Ripley B. and Ripley R. [9]. After removing duplicates and performing a screening of title and abstract, a total of 62 articles were considered.

**Figure 1:**
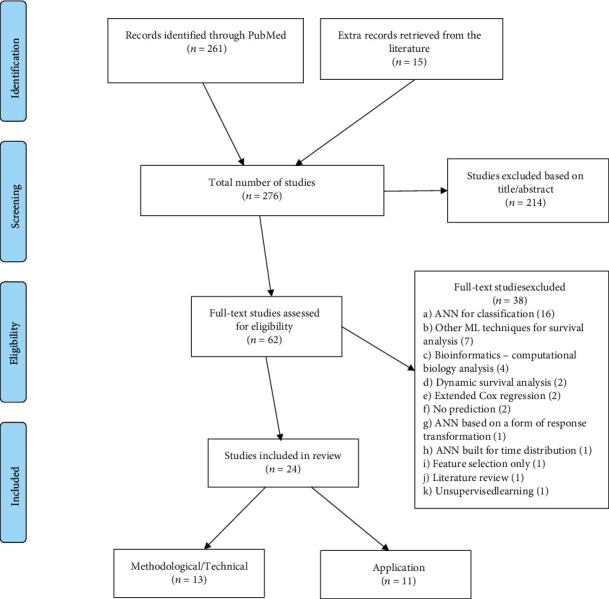
Preferred reporting items for systematic reviews and meta-analyses (PRISMA) flowchart. Reasons for exclusion of the 214 studies in screening step 1 were: ML techniques for classification (*n* = 98), predictions based on individual's images (*n* = 25), models with focus on feature selection (*n* = 18), bioinformatics/computational biology analysis only (*n* = 15), other ML techniques for survival analysis (*n* = 15), unsupervised learning (*n* = 10), other reasons (*n* = 33) including ML techniques for risk group stratification (*n* = 6), systematic/literature review (*n* = 6), new prediction tool (*n* = 5), ML techniques for regression (*n* = 4), ensemble of different ML techniques (*n* = 3), no prediction (*n* = 3), letter to the editor (*n* = 2), model for nonhumans (*n* = 2), models with focus on feature reduction (*n* = 1), and tutorial/case study (*n* = 1).

Our search strategy is summarized in Figure 1 as a Preferred Reporting Items for Systematic Reviews and Meta-Analyses (PRISMA) flow diagram [12]. We identified 24 relevant studies, 13 methodological, and 11 applications. Studies were considered eligible if they described the development of an SNN prediction model using prognostic factors, or its application (may build on an existing method to improve it to real-word medical data or simulation studies. We define an SNN prediction model as an ANN adapted to survival data and capable of making individual patient predictions with prognostic factors. We excluded studies that focused on other ML approaches, performed standard ANN classification/regression, used an ANN as an extension of Cox regression, or were solely concerned with feature selection/reduction. Applications involving nonhuman subjects, images (pathology images, magnetic resonance imaging, tumor slices, etc.) and computational biology analysis (e.g., predictions of gene expression) were disregarded. All nonoriginal articles (e.g., reviews and tutorials) were excluded. The reader can find the search string in PubMed and the detailed list of inclusion/exclusion criteria in the Supplementary Material (available here).

## 3. Methodologies

In this Section, we present the methodological approaches of neural networks for survival analysis in chronological order. The majority of the techniques were developed in the 1990s or early 2000s, followed by a long period with hardly any contributions in the field. Recently, the interest in the development of new methods has been rekindled, and modern approaches have been developed in specialized state-of-the-art software such as keras [13] in Python or R programming languages, which offer tremendous capabilities in modelling architecture and optimisers. Available options move beyond typical Feed Forward ANNs (FFANNs) and include deep learning and recurrent neural networks (RNNs), which were originally used only in nonmedical context, for example, for speech recognition and natural language processing.  Table 1 provides notations used in the manuscript.

### 3.1. Basic Components of Survival Neural Networks

Neural networks have a layered structure which is based on a collection of units (also called nodes or neurons) for each layer. The input layer picks up the signals and passes them to the next layer which is called “hidden” after the application of a (usually nonlinear) activation function. SNNs can have one or multiple hidden layers next to each other that connect with the previous layer. Signals are transmitted towards the output layer which is the last layer of units where desired predictions are obtained. For SNNs, the output layer predicts (conditional) event probabilities or survival probabilities. A bias unit is an extra node added to each preoutput layer that stores the value 1 (it allows the activation function to be shifted to left or right to better fit the data). Bias units are not connected to any previous layer. Connections between the artificial units of different layers are called edges. These have a weight which adjusts through training increasing or decreasing the strength of each connection's signal. The simplest type of a neural network is a FFANN where the information moves in only one direction—forward: from the input units to the hidden units (if any) and to the output units. Recently, more and more researchers build deep neural networks which are ANNs with multiple hidden layers between the input and the output layer. Recurrent neural networks are also a class of FFANNs where connections between units form a directed or an undirected graph along a temporal sequence (of time intervals).

There are two basic data formulations for right-censored survival data which is the main focus here. For some methodologies, the wide data format is sufficient (standard data format with a single line per patient). However, several methods require data transformation into a long format where each patient is replicated multiple times with the survival times being divided into a set of *k* nonoverlapping time intervals indicating months or years. Different terminologies such as prognostic variables, survival covariates, covariate vector, prognostic/clinical features, or predictors are used to denote the input units (features) of SNNs for the purpose of text enrichment. Note that some of the networks can include time-varying covariates (variables that change values over time during the follow-up period) as part of the input units if a methodology necessitates data transformation into a long format.

**Figure 2:**
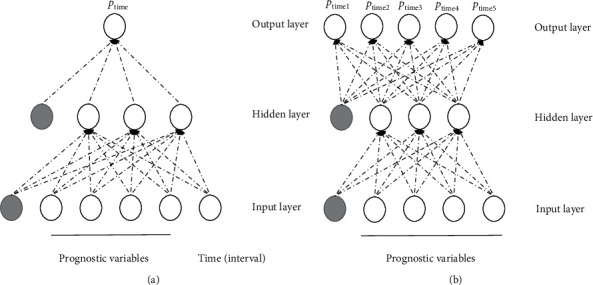
Two basic architectures of survival neural networks. (a) A network where time (interval) is coded as a prognostic variable (input feature). Data transformation into a long format is required for each patient. The output layer makes predictions in a given time interval. (b) A network where time (interval) is not coded as part of the prognostic variables. The wide data format is adequate for each patient. The output layer makes predictions at multiple sequential (nonoverlapping) time intervals.

An example of two basic architectures for SNNs is illustrated in Figure 2. These are FFANNs with one hidden layer. The network's architecture depends on whether the time (interval) is coded as part of the prognostic variables or not.

### 3.2. Early Methodological Approaches

The first attempt of modelling neural networks for censored data was made by Ravdin and Clark [14]. The authors use a simple 3-layer FFANN and code time as an additional prognostic variable. Input features are replicated for several time intervals [1, ⋯, *T*_max_] with equal event rates, where *T*_max_ is the maximum follow-up time (in years). A patient who experienced the event of interest, is replicated exactly *T*_max_ times, while a censored patient is replicated only until the time of censoring. The output layer contains a single output unit representing the survival status and is set to 0 for all time intervals where the subject is alive and to 1 for the time interval where the event of interest occurred (and the following intervals up to *T*_max_). The hyperbolic tangent activation function is used for the units in the hidden and output layers. To correct for the bias introduced by the data transformation in a long format (as the number of deaths is over-represented in the late intervals), a selective sampling approach is performed, such that the proportion of deaths matches the information of the Kaplan-Meier [8] estimate. Selective sampling, however, is not an exact procedure, and weighting cases would be a preferable approach [9]. The output layer provides death probabilities and can be seen as a prognostic index. An advantage of the methodology proposed by Ravdin and Clark is that time-varying covariates can be included, as subject entries are duplicated across multiple time periods.

De Laurentiis and Ravdin proposed two alternative FFANNs [15]. The first is very similar to Ravdin and Clark's approach and also specifies the time interval as an additional input variable. In this model, the distinct time intervals are selected such that each interval reflects a constant increase in event probability. Again, no data is present for censored cases after the last interval on study. Bias is controlled in a similar fashion, by obtaining the same frequency of censoring and events. The second FFANN proposed by De Laurentiis and Ravdin is a multiple time point model. This network does not require any modification of the wide data format and can accommodate only baseline characteristics and no time-varying covariates. The output layer is a vector with multiple output units (nodes) of *I*_*k*_ nonoverlapping ordered intervals and estimates event (death) probabilities. In the training, data censored cases can be imputed at given times of follow-up (e.g., by Cox regression) or, alternatively, these output units can be deactivated. This approach mimics a *k*-class classification problem.

In the same year, Liestol et al. proposed ANN generalizations of standard regression models used for survival analysis [16]. They constructed ANNs comparable to the 2^*nd*^ network proposed by De Laurentiis and Ravdin, with and without the hidden layer. These networks have *k* output units estimating hazard scores and are denoted as chain-binomial models. In principle, these networks can be viewed as a modification of Cox regression models, where the time axis has been partitioned into a number of disjoint intervals (grouped survival data). Such a model for the observed data may be specified via the conditional survival probability *q*_*k*_ = *P*(*T* ≥ *t*_*k*_|*T* ≥ *t*_*k*−1_), with *k* = 1, ⋯, *K*. To implement it in a shallow network (no hidden layers) with *K* output nodes, the following parametrisation *w*_1*j*_ = *w*_2*j*_ = ⋯ = *w*_*Kj*_ = *β*_*j*_ and *j* = 1, ⋯, *p* can be applied, where *w*_*kj*_ is the weight assigned to the connection between input node *j* and output node *K*. This implies that all connections arising from the same input node *j* have the same weight. Then, for the output nodes, the following function will be computed:

(1) $$\begin{array}{c} O_{k}\left(x;\beta ,w_{k0}\right)=g\left(\beta^{T}x+w_{k0}\right), \end{array}$$

with *x* as the input variable, *β* = (*β*_1_,⋯,*β*_*p*_)^*T*^, and *w*_*k*0_ as the weight from the bias node of the input layer. By applying a sigmoidal (logistic) activation function *g*(*x*) = exp(*x*)/1 + exp(*x*), we obtain an output *O*_*k*_, which corresponds to the event (death) probabilities *p*_*k*_ of the grouped version of the Cox model [4]. Applying the activation function *g*(*x*) = 1 − exp(−exp(*x*)), ensures estimation of event probabilities as in the grouped version of the Prentice and Gloeckler model [17]. The log-likelihood function of such a model corresponds to the negative error (loss) function:

(2) $$\begin{array}{c} E=-\underset{i}{\sum }\underset{k}{\sum }\left\{Y_{ki}\log\left(O_{k}\left(x_{i};w\right)\right)+\left(1-Y_{ki}\right)\log\left(1-O_{k}\left(x_{i};w\right)\right)\right\}, \end{array}$$

for an individual *i* = 1, ⋯, *n* of output unit *k* = 1, ⋯, *K* having covariate vector *x*_*i*_, and observed responses (target values) *Y*_*ki*_. This loss function is minimized with respect to *w*, the connection weight matrix, using a back-propagation algorithm. Liestol et al. suggested extensions to nonlinear and nonproportional ANNs, which would require dropping the weight constraint and adding a hidden layer to the previous shallow network. This would lead to an increase in the number of parameters. A nonlinear and nonproportional ANN introduced in this way could be more appropriate in dealing with prognostic factors of nonlinear and time-dependent effects.

Another attempt at adapting ANNs to survival data was made by Lapuerta et al.'s study [18]. Here, the output variable of the FFANN represents the time of occurrence of clinical coronary events. Time is divided into three 40-month periods plus an additional period in which no event occurred during the 120 months. The initial values for the output vectors denote event (1), no event (0), or censorship (as an unknown outcome with the symbol ?). To improve predictive ability, two separate networks are used to impute missing outcomes of early censored cases in each training set for the second period (40-79 months) and third period (80-120 months). Imputations are not performed in the test data. The authors create a predictor network where the output neuron with the highest value indicates the most likely outcome between four different classes. This approach might become cumbersome in terms of computational cost as it requires the use of multiple ANNs.

Street used a standard FFANN with the hyperbolic tangent activation function for the units in the hidden and output layers [19]. The output layer consists of 11 ordered categories, (0, 1], (1, 2], ⋯(9, 10] years, plus a final category denoting time of more than 10 years (in which the event did not occur). The network estimates the probability of disease-free survival up to a particular year, learning multiple classes in parallel. The output node is +1 as long as an individual is recurrence-free and -1 thereafter. Censored cases are incorporated directly in the training set using the probability that a patient will have disease recurrence before a certain time. The probability is obtained by employing a variation of the standard Kaplan-Meier method. Hereto, each censored individual may relapse at time *t*, given that no relapse has occurred at *t* − 1, and the disease-free survival time is used as the starting time (instead of time 0). Street uses the probabilities generated by the ANN to separate cases into those with “good” and “bad” prognosis and to estimate survival curves for individual patients. The author scales the probabilities to the range of the activation function by using activation = 2∗probabilities − 1 and specifying the relative entropy error function. Street's approach cannot be considered as a classification problem because of the many incomplete data cases (it is unknown whether an individual is recurrence-free for these instances).

**Figure 3:**
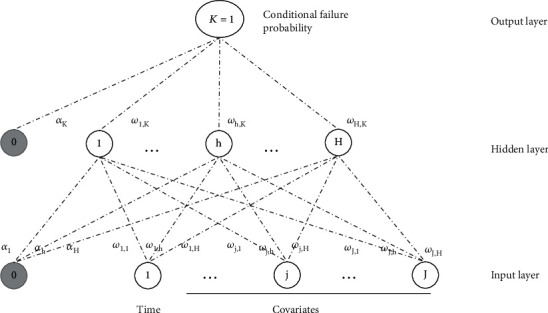
Visualization of the PLANN by Biganzoli et al. [20].

Biganzoli et al. introduced the partial logistic ANN (PLANN) [20]. This is a variation of the network proposed by Ravdin and Clark [14]. It has a single hidden layer, one unit (node) in the output layer, and uses the time indicator as an additional input variable. Each prognostic variable is replicated for the number of intervals until death or end of follow-up. A major difference from Ravdin and Clark's approach is that here patients are not included after the time interval of death.  Figure 3 shows a visual illustration of Biganzoli's PLANN. Nodes are represented by circles and the connections between them by dashed lines. The weights for the connection of the bias node with the hidden layer and the output layer are denoted by *α*_*h*_ and *α*_*k*_, respectively. The weights for the connections between input and hidden nodes and hidden and output nodes are denoted *w*_*jh*_ and *w*_*hK*_, respectively. The input layer consists of *J* nodes, given by the covariates, the time indicator, and a single bias node (0). The hidden layer consists of *H* nodes and one bias node (0). There is a single output unit (node) (*K* = 1) which computes conditional failure probabilities.

The output $\hat{y_{k}}$ of a PLANN with a single hidden layer for an individual *i* can be defined as:

(3) $$\begin{array}{c} \hat{y_{k}}\left(x_{i},w\right)=\phi_{o}\left(\alpha_{k}+\overset{H}{\underset{h=1}{\sum }}w_{hk}\phi_{h}\left(\alpha_{h}+\overset{J}{\underset{j=1}{\sum }}w_{jh}x_{ij}\right)\right), \end{array}$$

for *j* = 1, ⋯, *J* input nodes; *k* = 1 unique output node; *ϕ*_*o*_ and *ϕ*_*h*_ are the activation functions of the output and the hidden layer, respectively; *x*_*ij*_ represent the input value for an individual *i* and covariate *j*; *α*_*h*_ and *α*_*k*_ are the constant bias nodes for the input and the hidden layers, respectively. In general, *ϕ*_*o*_ will depend on the specified regression problem. For this SNN, Biganzoli et al. used the logistic activation function for both the hidden and output layer. FFANNs with logistic outputs (such as PLANN) can be regarded as flexible regression models for conditional probability estimation [21, 22].

To enable inclusion of covariates, Cox proposed the proportional odds model [4] for grouped survival times. The formula below shows that discrete hazard rates can be modelled using a logistic regression model:

(4) $$\begin{array}{c} h_{l}\left(x_{i}\right)=\frac{\exp\left(\theta_{l}+\beta^{T}x_{i}\right)}{1+\exp\left(\theta_{l}+\beta^{T}x_{i}\right)}, \end{array}$$

where *θ*_*l*_ = log(*h*_*l*_(0)/1 − *h*_*l*_(0)) of *l* = 1, 2, ⋯, *L* disjoint intervals *A*_*l*_ = (*t*_*l*−1_, *t*_*l*_] with *t*_0_ = 0 and *l*_*i*_ the interval of observation for the *i*^*th*^ subject.

PLANN is a generalization of partial logistic regression. The output values provide smoothed estimates of discrete hazards *h*_*l*_(*x*_*i*_, *a*_*l*_) for the midpoint *a*_*l*_ of the time interval *A*_*l*_. The survival is estimated as *S*(*t*_*l*_) = ∏_*l*=1_^*L*^(1 − *h*_*l*_(*x*_*i*_, *a*_*l*_)). The error function of the model, for a given individual, *i*, is defined as:

(5) $$\begin{array}{c} E\left(x_{i},a_{l}\right)=-\overset{n}{\underset{i=1}{\sum }}\overset{l_{i}}{\underset{l=1}{\sum }}\left\{\delta_{il}\log\left(h_{l}\left(x_{i},a_{l}\right)\right)+\left(1-\delta_{il}\right)\log\left(1-h_{l}\left(x_{i},a_{l}\right)\right)\right\}, \end{array}$$

with *δ*_*il*_ as the event indicator (1 at the interval of the event of interest and 0 otherwise). This error function is equivalent to the cross-entropy error function and to Equation (2). A weight decay penalty term is added to the weights in Equation (5) to avoid overfitting (*E*^∗^ = *E* + *λ*∑*w*^2^, regularisation *L*_2_) .

Biganzoli et al. used PLANN for flexible modeling of the hazard function of different cancer datasets, in an explanatory analysis. This approach has several favourable characteristics, including the presence of an analytical mathematical formulation, monotonicity of the survival curves, and the option to include time-varying covariates, as the neural network is fitted to data that has been transformed to long format.

### 3.3. Approaches at the Beginning of New Millennium

Lisboa et al. extended the PLANN approach in 2003 by introducing a Bayesian framework with automatic relevance determination (ARD) [23]. This approach, called the PLANN-ARD, was inspired by David Mackay's review of Bayesian supervised ANNs [24]. PLANN-ARD is robust in estimating weight parameters and carries out model selection, via regularization included within a Bayesian framework which consists of a sequential 3-step approach:

1. A penalty term, *L*(*w*, *k*), is added to the objective function in Equation (5) (similar to weight decay) where *k* is a set of Bayesian regularization parameters. The penalized objective function is *S*(*w*, *k*) = *E* + *L*(*w*, *k*)
2. Regularization parameters are estimated to control the penalty term
3. Model selection is performed by interpreting the evidence in favor of candidate networks (hyperparameter selection)

For tuning the hyperparameters, the empirical Bayes approach is preferable to cross-validation, as the latter is frequently very computationally intensive. PLANN-ARD soft-prunes irrelevant variables to carry out model selection (as part of the Bayesian framework). The authors suggest that this methodology can be more efficient in the allocation of patients into prognostic groups compared to the Cox model.

Given that enough hidden units are specified, ANNs can approximate any functional relationships (i.e, interactions between covariates) [25, 26]. Ripley R. et al. proposed two more discrete-time FFANNs [27]. Here, time is split into five nonoverlapping time periods (*I*_1_ : (0, 1], *I*_2_ : [1, 2), *I*_3_ : [2, 3), *I*_4_ : [3, 5), and *I*_5_ : [5, ∞)). No multiple records (repeated entries of the same individual in the data) are needed for these approaches.

For the first network, the likelihood is calculated by ∏_*i*=1_^*N*^∑_*k*=*m*_*i*_+1_^*l*_*i*_^*p*_*ki*_, where *m*_*i*_ is the last time period the *i*^*th*^ patient is known to have survived without relapse, *l*_*i*_ is the final time period during which the patient may have relapsed, and *p*_*ki*_ is the probability that the *i*^*th*^ patient relapses in time period *k*. Ignoring the ordering of time periods, the model can be estimated as:

(6) $$\begin{array}{c} \log\left(p_{k}\right)-\log\left(p_{1}\right)=\eta_{k}\left(x\right)\left(k=2,3,4,5\right), \end{array}$$

with *η*_*k*_(*x*) = *y*_*k*_ − *y*_1_ using an ANN with the softmax activation function for the units of the output layer. The probabilities are computed as *p*_*k*_ = exp(*y*_*k*_)/∑_*l*_exp(*y*_*l*_) (softmax formula) where *y*_*k*_ are outputs of the network.

The second network relies on more complex methodology which incorporates ordinal outcomes. This ANN has a single output unit to model the function *η*, which is now independent of the output class *k*. The cumulative event probabilities, *γ*_*k*_ = *F*(*t*_*k*_|*x*), are modelled as:

(7) $$\begin{array}{c} \log\left(\frac{\gamma_{k}}{1-\gamma_{k}}\right)=t_{k}-\eta \left(x\right)\;\left(k=1,2,3,4\right), \end{array}$$

where *t*_*k*_ indicates the end of the *k*^*th*^ time period. Constraints on *t*_*k*_ : *t*_1_ ≤ *t*_2_ ≤ *t*_3_ ≤ *t*_4_ are set to ensure that *γ*_*k*_ are increasing (ordinality of outcomes).

### 3.4. Modern Methodological Approaches

Deep learning ANNs are frequently used for prediction of output features, especially in the context of image classification [28, 29]. Applying deep learning methodology to medical survival data, however, poses the risk of overfitting, as the available sample sizes are typically small. Matsuo et al. predicted survival by using a deep neural network (DNN) with a hierarchical structure and FFANNs in the first layers of the model [30]. The DNN contains 2 subnetworks with fully connected layers to jointly optimize the C-index and Mean Absolute Error (MAE). For each subnetwork, the optimization is performed separately. The C-index quantifies the probability that the predicted event times of two randomly selected individuals have the same order as their true event times. Due to the presence of censored data, not all pairs can be compared; this implies that a pair of subjects are comparable if the earliest time is an event or both are events. The C-index is a measure of probability of concordance between the observed and the predicted survival. The MAE is defined as the absolute difference between the observed survival time and the survival time predicted by the subnetwork. The authors found that the DNN performance improved on inclusion of more clinical features (input variables). A drawback of DNNs is that they are frequently computationally intensive and can be too complex for clinical insights.

Bora et al. developed time-binned neural networks to predict recurrence-free survival of non-small-cell lung cancer after surgery, using 30 clinico-pathological features [31]. The authors present one supervised learning binned-time survival analysis model (called su-DeepBTS) and one semi-unsupervised learning model (called su-DeepBTS). Here, we focus only on the supervised learning model su-DeepBTS. This is a shallow network where the output layer provides the survival probability in each predefined time interval (recurrence-free survival in months). The output value, *y*_*j*_, is 1 when a patient is alive without relapse at the beginning of the *j*^*th*^ time interval *I*_*j*_, and 0 after relapse. For censored patients, *y*_*j*_ is 1 until a patient is lost to follow-up and ∏_*i*=*t*_*i*_≤*I*_*j*__(1 − *d*_*i*_/*n*_*i*_) after censoring occurs (Kaplan-Meier survival probability), where *n*_*i*_ is the total number of samples without recurrence at the beginning of the *j*^*th*^ time interval, and *d*_*i*_ is the number of events. The activation function of the output layer is the sigmoid (logistic). The root mean squared error (RMSE) between the true *y*_*j*_ and the predicted ${\hat{y}}_{j}$ is used as loss function.

#### 3.4.1. Survival Recurrent Networks

Oh et al. use a survival recurrent network to train time-sequential outcome data for gastric cancer patients [32]. Their model is a DNN containing four recurrent neural network (RNN) layers in a total of seven layers, with the number of nodes gradually reduced across hidden layers. This network takes as inputs patient prognostic features and the survival probability of the previous year. In the following year, a comparison is performed between the predicted and observed survival probabilities. Survival for each time interval is denoted as either 1 (alive), 0 (dead) at the time of observation, or, for censored cases, as a ranking score in between 0 and 1. The predicted survival probability is updated every year with a weight, which is a tuneable parameter. The input layer consists of 25 prognostic features plus two survival features. The output layer consists of two nodes and is activated with the softmax function. As part of the procedure, variables of an individual are embedded (categorical variables are mapped to a vector of continuous numbers) for purposes of dimensionality reduction. This ANN approach to modelling survival data is complex which means it could lead to a poor generalizability on new data (overfitting training data) and/or a less intuitive interpretability of results.

**Figure 4:**
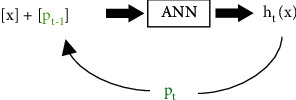
A schematic representation of the SNN by Han et al., adapted from [33], built for 242 patients with synovial sarcoma. Here ANN means artificial neural network, *x* is the set of 9 clinical features, *p*_*t*−1_ is the survival probability of the previous year *t* − 1 sequentially updated (10^*th*^ input feature), *h*_*t*_(*x*) is the predicted survival risk (alive/death probability), and *p*_*t*_ the predicted survival probability for the following year *t*.

A comparable learning algorithm was developed by Han et al. [33]. Han et al. describe a deep learning based survival model that can analyze patients lost to follow-up in a sequential manner (Figure 4). The network contains an input layer, 3 hidden layers with the number of nodes reduced across the layers, and an output layer. Information is updated every year. It is composed of three learning systems: nine clinical features *x*, the survival probability *p*_*t*−1_ for the previous time of follow-up sequentially updated (10^*th*^ input feature), and nonparametric ranking scores 0 < *r* < 1 for censored cases. Each time the ANN predicts the survival probability for the following year *p*_*t*_. The recurrent loop reinforces training of the network sequentially, updating the residuals *λ* between the real outcome *Y* (1 = alive and 0 = dead) and the probability $\hat{Y}$, predicted by the SNN. A modulating parameter connects the residuals with the survival probabilities. As in Oh et al.'s network, the output layer contains two nodes that are activated with the softmax function, which represent the predicted alive/death probability. This DNN might be biased because individuals who survive longer will be used more times for retraining, resulting in connection weight matrix, *w*, optimized for longer survivors.

Sung et al. developed RNNs with long short-term memory (RNN-LSTM), with the purpose of performing a risk classification for the prevention of cardiovascular disease, using national time-series health examination data [34]. This model includes a large number of patients (361239), randomly sampled in South Korea. The authors transform the binary output variable into multiple time-point output vectors for specific time-point analysis. The output layer includes yearly intervals from 2 to 10 years. The RNN-LSTM estimates the probability of survival for each interval. To take into account censored individuals, the probability of disease is estimated using Kaplan-Meier methodology. This network can incorporate time-varying covariates, and, in this application, provides more accurate predictions than the Cox model, suggesting such an approach may be well-suited to time-series data in particular.

## 4. Applications

In our search, we identified eleven applications, of which eight used real data and three used simulated data to investigate model behaviour in different scenarios. In some of these studies, the original methods were modified to improve prediction. Furthermore, as interpretability of results is crucial for clinical decision making, some studies focused on extracting interpretations from the ANNs (often called “black boxes” as they do not provide insights on the structure of the function they approximate). The applications make use of different performance measures, which is likely due to the dynamic evolution of the field over the last decades.

Xiang et al. [35] compared three different approaches in a simulation study. Nine data designs were simulated with 2 or 4 covariates, various censoring patterns, interaction between covariates, as well as proportional or nonproportional hazards. Survival times were generated using inverse probability transformations (details in the paper). For the purposes of this review, we only consider the SNN developed by Liestol et al. [16] as the other two networks do not meet our search inclusion criteria. Time was divided into three distinct intervals, in which the hazard was assumed to be constant. The authors chose the general form of the method (no proportional hazards, dropping the weight constraint: see Section “sec:methodologies-early-methodological-approaches”). A simple FFANN with one input, one hidden, and one output layer was developed. The quasi-Newton algorithm was used to minimize the negative log-likelihood. The performance of the SNN varied according to 9 underlying data designs, but none outperformed the Cox regression model. Kattan [36] applied the same methodology to 3 large urological datasets. For this study, the author preserved proportional hazards for the network (by applying weight constraints). The author claims that, although theoretically attractive, ML techniques often do not result in an improved prediction accuracy.

Chi et al. [37] applied the SNN developed by Street [19] to two breast cancer datasets. The FFANN had three layers with sigmoid activation functions. It predicted the disease-free survival probability for each time unit. A slight modification was made to the labelling of the output vectors, using +1 up to recurrence time and 0 thereafter. The authors concluded that ANNs can successfully predict the probability of disease recurrence.

The PLANN-ARD Bayesian framework has been used several times for prediction in medical studies. Jones et al. applied PLANN-ARD to data on patients with laryngeal squamous carcinoma [38], in which 97.9% (855 out of 873) died from the disease. When comparing the SNN to a Cox model, the authors found that the SNN performed better in separating patients' survival based on dichotomous variables. Taktak et al. performed a double-blind multicentre study for uveal melanomas [39]. They applied a PLANN-ARD, using 5-fold cross-validation to tune the hyperparameters instead of an empirical Bayes approach. A Bayesian mechanism was used to compensate for skewness in the data vector, resulting from the necessary data replication when transforming the data to long format [23]. The authors found a better performance of the SNN when compared to the semiparametric Cox model and other models (the log-normal model, the partial spline model, and the partial logistic radial basis function network).

Five years after the development of PLANN-ARD, Lisboa et al. [40] applied the approach to breast cancer data. They extended the existing methodology to a competing risks model, where the two competing events are disease-free survival and breast cancer related mortality. This SNN provided a smoothed estimate for the hazards over time (assumptions about proportionality not required). The Bayesian framework for variable selection was extended to allow for continuous variables. To evaluate performance, a time-dependent C-index was used, which is an extension of the Area Under the Receiver Operating Characteristic (AUROC) measure [41]. The authors concluded that PLANN-ARD was a useful tool for risk assessment, as it distinguished high and low risk patients better than the Cox model.

Amiri et al. applied a hierarchical ANN for risk assessment of gastric cancer patients [42]. Input features consisted exclusively of binary covariates. The network was a simple feed-forward with three nodes in the hidden layer, which computed the probability of survival in different periods. The authors observed that the SNN had a smaller mean standard error for the survival probabilities than the Cox proportional hazards model. They noted, however, that the baseline survival of the SNN may be unreliable as a consequence of the small sample size (*N* = 330) of the study.

Biglarian et al. compared the PLANN method with Cox models in a simulation study [43]. Percentage of censoring was chosen between 20.0 and 80.0% and the data were simulated with linear and nonlinear effects for the hazards. Model hyperparameters were tuned (a set of parameters was identified that leads to best performing model in the training data), using the Bayesian information criterion (BIC). Model fit was assessed in the test set, using the mean squared error (MSE). This study concluded that prediction accuracy in more complex datasets depends on the level of censorship. Use of PLANN was suggested for data with a high percentage of censoring and for modelling complex interactions.

Spelt et al. applied PLANN to predict long-term survival after liver resection of metastases, for patients with metastatic colorectal cancer [44]. The model was an extension of the network by Biganzoli et al. [20] and used an ensemble of SNNs. Training and validation were performed using 5-fold cross-validation, applied to 20 slightly different datasets, which were created by performing multiple imputations of missing values on the original data. The networks were combined within a single prediction model. The output of the ensemble was the mean output of all individual SNNs. Harrell et al.'s C-index was used as an performance measure [45]. Building on the work of Lippmann and Shahian for odds ratios [46], time-dependent hazard ratios for each variable in the SNNs were provided. Prognostic variables were ranked and minimized for the trained SNN. Order of variable relevance was obtained by measuring the change in baseline C-index (model with all variables) after removal of each of the risk factors, one at a time.

Gong et al. investigated the PLANN approach in a simulation with a view to the field of pharmacometrics [47]. As in the study by Biglarian et al. and Gong et al. investigated both different proportional hazard functions (linear, nonlinear) and different censoring percentages. To interpret the results, the authors employed the connection weights algorithm proposed by Garson [48] to calculate the relative importance of each input variable, and evaluated this method in a high-dimensional setting. Performance was assessed using the C-index, and the authors found that PLANN outperformed Cox regression. PLANN was less sensitive to changes in sample size and censoring percentage than Cox regression, and achieved the best performance when predictor variables assumed nonlinear relationships in the hazard function. Additionally, for high-dimensional simulated data, PLANN was able to identify all predefined influential variables.

Kantidakis et al. compared PLANN with Cox models for large liver transplantation data (*n* = 62294 patients, 97 predictors). The authors described novel extensions to existing PLANN architecture (i.e, hyperparameters, activation functions, and time interval specification) [49]. The extended PLANNs were tuned with the Integrated Brier Score (IBS) as the main criterion, which is a global summary of Brier score over the whole range up to the time horizon of the study (10 years) [50, 51]. The SNNs showed better performance than the Cox models based on IBS at 10 years, and the extended PLANN with 1 hidden layer was as calibrated as the Cox model with all variables (the predicted survival probabilities were similar to the observed survival probabilities estimated by using Kaplan-Meier's methodology). Emphasis was given on the advantages and pitfalls of each method and on the interpretability of the ML techniques. As in Garson, the connection weights algorithm [48] was used to identify the strongest prognostic factors.

## 5. A Critical Perspective

In this Section, we critically appraise relevant characteristics of the 13 methodological and the 11 application studies selected for this review (details on Figure 1). Excel sheets were constructed (available in the online version as pdf files) that list the relevant prediction model characteristics. Additional information is provided in the Supplementary Material (availanle here) (overview of the extracted items in each study, 9 tables regarding the study characteristics) .

### 5.1. General Study Characteristics

Of the 24 studies, 21 (87.5%) made use of existing data, while three (12.5%) applied the methods to simulated datasets. Descriptive statistics are shown in Table 2. The median total sample size was 920 patients, the median number of predictors was 7 (low-dimensional data), and the median percentage of censoring was 70.8% (10 of 24 studies considered multiple outcomes). Medical applications were mainly in the field of oncology (73.5%, 25 datasets). The majority of these studies conducted research on breast cancer (10 datasets), cervical cancer, gastric cancer, or prostate cancer (2 datasets each). Other fields of application comprised cardiovascular disease, coronary artery disease, liver transplantation, and postpartum amenorrhea (2, 1, 1, and 1 datasets, respectively).

Clinical endpoints of interest included overall survival (analysed 16 times, 47.1%) and disease-free (or progression free, recurrence-free, relapse-free) survival (analysed 12 times, 35.3%). Remaining endpoints were breast cancer specific mortality (5.9%), death or hospitalization due to cardiovascular events (5.9%), menstruation-free survival (2.9%), and time to clinical artery events (2.9%).

The strategy used to address the missing data (if any) was unclear for 9/21 (42.9%) studies (disregarding the 3 simulation studies that did not contain any missing data). Single or multiple stochastic imputation was used for 6 studies (28.6%) and ad hoc approaches (separate attribute or mean/median imputation) were used in 5 studies (23.8%). One dataset had no missing data. Ad hoc approaches to missing data can be problematic, as they can alter the distribution of a variable (if there is a substantial number of missing values). Multiple stochastic imputation, which replaces each missing value with multiple plausible values, is the preferable option [52], as the variability in multiple predictions reflects the uncertainty of the imputation process. It is understandable that multiple imputations may not be considered due to computational cost. Nevertheless, a single stochastic imputation is still superior to an ad hoc fix, since imputation algorithms are more likely to preserve the original data structure. Examples of such algorithms are *k*-nearest neighbor and random forest (missForest) [53, 54].

### 5.2. Model Development

Different aspects of model development for SNNs were considered: (1) whether the hyperparameters were tuned and which was the performance criterion for model development; (2) how the prognostic variables were scaled; (3) which programming language was used.

Hyperparameters are fundamental to the architecture of an ANN. They fine-tune the performance of a prediction model, preventing overfitting and providing generalizability of the model to new “unseen” data. Choice of hyperparameters can be a challenge in the modern era of building SNNs with state-of-the-art software that allows for numerous choices. Commonly tuned parameters were penalty terms in the likelihood (e.g., weight decay) and the number of units (nodes) in the hidden layer(s). In the majority of studies (15, 62.5%), the approach to training hyperparameters was unclear, with 6 of these studies (25.0%) failing to report whether parameters were tuned or default values were chosen. In 4 studies (16.7%) parameters were tuned, in 3 studies (12.5%) some parameters were tuned and some were assigned default values, while in 2 studies (8.3%) default values only were chosen for the hyperparameters. The performance criterion for model development (hyperparameter tuning) was examined across the 24 studies. The training criterion was unclear for 6 studies (25.0%). For 5 studies (20.8%), neural network hyperparameters were trained based on the log-likelihood, for 3 studies based on the C-index (12.5%), and for 2 studies (8.3%) based on the Area Under the Curve (AUC). Other criteria used for model development are provided in the Supplementary Material (availanle here). Better reporting of the choice of hyperparameters (which parameters were selected) and of the training procedure (how they were tuned) is needed. This will help researchers to better understand how the model was developed and will facilitate reproducibility.

In ANNs, input features are typically scaled to ensure that all features have a comparable scale, which allows an update of the same rate, resulting in faster algorithm convergence. The procedure was unclear in 10 of the 24 studies (41.7%), scaling was unnecessary in 7 studies (29.2%), and normalization (minimum and maximum values of features are used for scaling) was applied in 5 studies (20.8%). Standardization (mean and standard deviation of features are used for scaling) was applied in only 2 studies (8.3%). A precise description of the scaling approach (normalization or standardization) should be provided by researchers.

The programming language used for the development of the ANN was unclear in 7 studies (29.2%). Python was employed in 4 (16.7%) and R in 2 (8.3%) of the more recent studies. In the previous decades, MATLAB was used 3 times (12.5%), NeuralWare 3 times (12.5%), S-plus 3 times (12.5%), while Epilog Plus and PlaNet were used 1 time each (4.2%). There is a trend towards employing Python, utilizing the keras and Theano libraries, which can build state-of-the-art ANNs with multiple options for layers, optimisers, and error (loss) functions. These two libraries also have an interface available to the R programming language. It is strongly encouraged to share code developed for new methodologies or applications of existing methodologies in publicly available repositories (e.g., GitHub) to support reproducability and good clinical practice.

### 5.3. Model Validation

We examined the validation approach for each of the 34 outcomes (clinical endpoints of the studies). Single random split was used 17 times (50.0%), with the data split into single train-test or train-validation-test parts. When the data are split into train-test parts, the best model for training data is chosen based on model's performance on test data, whereas when the data are split into train-validation-test sets, the best model for training data is selected based on the performance of the model on validation data. Then the test data are used to internally validate the performance of the model on new patients. Resampling (cross-validation or nested cross-validation) was used 9 times (26.5%). External validation (testing the original prediction model in a set of new patients from a different year, location, country, etc.) was used 4 times (11.8%). External validation involved the chronological split of data into training and test parts 3 times (temporal validation) and validation of a new dataset 1 time. Multiple random split was used 2 times (5.9%), with the data split into train-test or train-validation-test data multiple times. Validation was not performed for 2 datasets (5.9%). We recommend reporting the steps of the validation approach in detail, to avoid misconceptions. In case of complex procedures, a comprehensive representation of the validation procedures can be insightful. Researchers should aim at performing both internal and external validations, if possible, to maximize the reliability of the prediction models.

Table 3 shows the performance measures used for model validation in the 24 studies. A popular measure in the survival field, the C-index, was employed in 8 studies (33.3%, as C-index or time-dependent C-index) and AUC in 5 studies (20.8%). Notably, during the screening process, several manuscripts were identified, where AUC and C-statistic were used interchangeably. While there is a link between the dynamic time-dependent AUC and the C-index (the AUC can be interpreted as a concordance index employed to assess model discrimination) [55], the two are not identical and some caution is required. Apart from the C-index, there was no other established measure in the 24 studies (large variability). This issue is of paramount importance as validation (and development) of the SNNs depends on a suitable performance measure. Any candidate measure should take into account the censoring mechanism. By employing performance measures that are commonly used in traditional classification ANNs, such as accuracy, some SNNs were suboptimally validated. Consistency in the use of performance measures should also be considered. In the simulation study of Biglarian et al. [43], hyperparameter values for PLANN were based on the Bayesian information criterion (BIC), while validation of the SNN performance on the test data was performed using the mean squared error (MSE), and the comparison with Cox model was based on the C-index. Proper measures should be employed for model development and validation of time-to-event data (see the book of van Houwelingen and Putter [5]).

Reporting of confidence intervals for the predictive measures was examined; 13 studies (54.2%) did not provide confidence intervals. Repeated data resampling was practiced in 6 studies (25.0%). The following remaining approaches were observed: repeating the simulations 500 times, rerunning the SNN 10 times for each covariate, and using a nonparametric confidence interval based on Gaussian approximation (4.2% each). The method of choice was unclear in 2 studies (8.3%). There is a strong need for the development of methods which reflect the amount of uncertainty of an evaluation criterion. This would provide additional insights into the predictive accuracy of the model.

Another important aspect of a prediction model is calibration. It refers to the agreement between observed survival probabilities estimated with Kaplan-Meier's methodology and the predicted outcomes. Typically, a plot is produced where the subjects are divided into 10 groups based on the deciles of predicted probabilities. Observed survival probabilities are plotted against predicted. In this review, calibration plots were available for only 11 studies (45.9%). Calibration of the SNNs was not assessed in most studies, and as such a neutral comparison with the Cox proportional hazards model could not be established. This is in accordance with the findings of Christodoulou et al. [56], which pinpoint an urgent need for more attention in calibration of modern ML techniques versus traditional regression methods to achieve a fair model comparison in the classification setting.

### 5.4. Comparison with Cox Model's Performance

The Cox proportional hazards regression model assumes proportionality of hazards across different prognostic groups over time. Any interaction between predictors and/or time needs to be manually specified by the user (e.g., fractional polynomials and splines). This may be difficult when a large set of prognostic factors is available. ML techniques such as ANNs, which are flexible and data-adaptive, relax this assumption and can naturally incorporate multiway interactions between the input features. This characteristic together with the rise of computational power and the collection of large-volumes of data (with electronic healthcare records) has contributed to the popularity of ANNs. However, the Cox model remains the most common choice for survival data. Therefore, any new prediction model including SNNs should be compared to the traditional Cox model to be considered in clinical practice.

Of 24 studies, 19 reported comparisons between Cox models and SNNs. We assessed whether interaction terms were specified in the models to obtain optimal predictive performance in Cox regression. Fifteen studies (78.9%) did not consider interaction terms between the predictors, information was unclear for 2 studies (10.5%), and 2 simulation studies considered interaction terms when applicable (10.5%). This result suggests suboptimal attention to the development of Cox models, which in turn undermines inferences made regarding comparative SNN and Cox model performance. For datasets with a large number of prognostic factors (*p* > 10), a number of interaction terms can be selected based on external knowledge and clinical expertise (see [6]).

Secondly, the author's claim for the performance of SNN was investigated. Among the 19 studies comparing SNN and Cox model's performance, 9 (47.4%) claimed better predictive performance of the SNN, while 5 reported a similar or better performance (26.3%) of the SNN compared to the Cox model. The performance was similar to Cox's model in 5 studies (26.3%). These result may be influenced by publication bias, as articles with favorable results are more likely to be published than articles with poor results.

A fair comparison between SNN and Cox model approaches to modelling survival data should include model validation with proper evaluation measures, a comparison of calibration curves, and the inclusion of nonlinear terms and interactions for Cox models, where applicable and possible. On the preface of his textbook on clinical prediction models, Steyerberg reflects on exaggerated claims of modern method performance, which are lacking in convincing presentation of evidence and frequently involve suboptimal strategy choices for the regression model competitor [57].

## 6. Discussion

To the best of our knowledge, this is the first ever attempt at a large-scale review of SNNs in medicine using prognostic factors (1st January 1990–31st August 2021). It included 24 studies (13 methodological and 11 applications), where ANNs were employed for time-to-event prediction with right-censored data, mainly in the field of oncology (73.5%, 25 datasets) with a particular focus on breast cancer research (10 datasets). This might be due to the fact that survival analysis is well-suited to long-term outcome prediction (e.g overall survival), which is of primary interest in the field of oncology. Several methodologies were developed in the 1990s and were in later years applied to more complex datasets for clinical prediction. The majority of the SNNs were simple FFANNs, with the exception of some recent publications, which made use of deep ANNs and Survival Recurrent Networks. Amongst the methods used, two general trends can be distinguished: networks with a unique output unit and a time indicator variable added as an extra input feature and networks with multiple outputs representing *k* nonoverlapping time intervals. The former approach requires that the data are replicated multiple times, for each of the time intervals considered, and allows for the incorporation of time-varying covariates.

We excluded studies where SNNs were built for bioinformatics/computational biology analysis, dynamic survival analysis, focused on ANN extensions of the Cox model, studies that did not evaluate model performance, or where predictions were based on individual's images (pathology images, magnetic resonance imaging, tumor slices etc.) (see section “sec:conducting-the-review”). We addressed this review in a pragmatic way using the biomedical database PubMed and focusing on SNNs for prediction using prognostic factors. We acknowledge that we may have missed some articles during the process. Below, we briefly summarize some other important methodological developments of the last three decades.

Faraggi and Simon [58] extended the Cox model by replacing the linear function *β*^*T*^*x*_*i*_ with the output *ϕ*_*o*_(*x*_*i*_, *w*) of an ANN with logistic hidden and linear output layers. No bias unit is specified for the output layer, and the model is subject to the proportional hazards assumption. A modern deep survival analysis approach related to Faraggi and Simon's work was described by Katzman et al. [59]. Here, the authors construct a deep FFANN where the output of the network is a single unit which predicts the log-risk function and can be used to extend Cox regression (DeepSurv; an open source Python module). DeepSurv provides personalised treatment recommendations and is capable of predicting the effect of a specific patient's characteristics on the risk of failure. A practical extension of such work could involve the use of convolutional neural networks on medical imaging data for risk prediction (out of scope here). Very recently, a multilayer deep learning Cox-based prediction model (another extension of the linear function *β*^*T*^*x*_*i*_) was proposed by Sun et al. [60] for high dimensional survival data in a genome wide association study and was also applied by Hao et al. [61] in ultra-high-dimensional genomic data (number of predictors >10^5^). It is shown that it cannot only outperform several existing survival prediction models (Random Survival Forest, Cox LASSO, Cox Ridge) in terms of accuracy but also detect clinically meaningful risk subgroups by effectively learning the complex structures among genetic variants.

Biganzoli et al. extended the PLANN methodology to competing risks (PLANNCR), in a study of primary invasive breast cancer [62]. PLANNCR is an ANN for the joint modelling of discrete cause-specific hazards and can be used for both discrete and grouped survival data. The output layer contains multiple nodes (competing risks) that estimate discrete conditional event probabilities. PLANNCR uses logistic and softmax functions for the hidden and output layer, respectively. The error function that is minimized corresponds to the multinomial likelihood. The degree of smoothing for output nodes is modulated by the number of hidden nodes and the penalization of the error function (weight decay in the loss function). PLANNCR can be implemented using standard ANN software that is able to accommodate multiple classification. Lisboa et al. published an ARD extension of PLANNCR (PLANNCR-ARD) [63]. The authors apply the methodology for local and distal recurrence of breast cancer, in an approach that requires no prior domain knowledge and performs model selection within a Bayesian framework. Kantidakis et al. performed a simulation study [64] to compare the predictive performance of PLANN original [20] and PLANN extended (1 hidden layer) [49] with Cox models for noncomplex clinical data (small/medium sample size, low dimensional). Methods were compared for scenarios where different percentages (20%, 40%, 61%, 80%) of censored data were present. ML and Cox models showed similar predictive performance on simulated data for most scenarios. C-index, Brier score, or Integrated Brier Score were used for the comparison. Results of this study show that the statistical models were often better calibrated.

Fornili et al. presented a simple FFANN for the purpose of analyzing disease dynamics in a survival analysis context [65]. This SNN, applied to breast cancer data—specifies, for the output unit, the smoothed hazard as a function of time interval and prognostic factors. This approach is known as Partial Exponential ANN (PEANN), a nonlinear extension of generalized linear models for right-censored survival data [66] and a direct extension of the PLANN method for piece-wise data. The network uses the logistic and the exponential functions for the hidden and output layers, respectively. Such method is best-suited to modelling the hazard shape of diseases with a long follow-up and allows for the exploration of nonlinear and nonadditive effects.

Ching et al. developed Cox-nnet [67]—a new ANN framework for patient prognosis using transcriptomics data. This FFANN has an input layer, one fully connected hidden layer with 143 nodes (set as the square root of more than 20000 input features) and one output “Cox regression” layer. To avoid overfitting, different regularization methods are employed, such as ridge (weight decay), dropout, and a combination of ridge and dropout [68]. The author compared an ANN with no hidden layer (shallow), a single hidden layer, and two hidden layers, and found that a single layer neural network had the best performance based on C-index.

Very recently, two novel deep learning approaches have been published for dynamic survival analysis. Changhee et al. proposed Dynamic-DeepHit for longitudinal and time-to-event data with competing risks to issue dynamically updated survival predictions for cystic fibrosis patients [69]. This network is trained by leveraging a combination of loss functions that capture the right-censoring, and the associations of longitudinal measurements with disease progression. It provides a remarkable improvement in discriminating individual risks of different causes of failure. This model can also provide useful clinical insights by identifying covariates which are influential for different competing risks (risk predictions interpretation). In the same year, Jarrett et al. developed temporal convolutional networks for Alzheimer's disease (called MATCH-Net) [70]. This CNN is designed to capture temporal dependencies and heterogeneous interactions in covariates and patterns of missingness for personalised risk prognosis. Its performance is compared with statistical and deep learning benchmarks showing incremental sources of gain from various design choices.

A critical appraisal was carried out to pinpoint current limitations and identify future research directions. Our findings are summarized in Table 4. Based on these findings, we make the following recommendations. Complete and transparent reporting of modelling steps and analysis is necessary (e.g., more details on training and test data) to enable reproducibility and to allow critical appraisal of the results by a wider audience [71, 72]. In the event of missing data, a single or multiple imputation approach should be used, prior to SNN development (see also section “subsec:general_study_characteristics”), to avoid discarding patients from nearly complete records. Hyperparameter selection and training should be more extensive with the performance criterion for model development clearly reported. Careful tuning of parameters can prevent overfitting and improves the generalizability of the prediction model. When developing an SNN, the following elements must be considered: the number of hidden nodes, the penalty terms, the activation functions, and the optimizers. Of particular importance is the choice of performance measure for model validation, which we observed to be sometimes poorly chosen (see section “subsec:model_validation”). A suitable performance measure should take into account the censoring mechanism (see the book of Houwelingen and Putter [5]). Additionally, model calibration should be assessed, preferably through calibration plots. In the studies of our review, the median sample size was 920 patients, and the median number of predictors was 7 (low-dimensional data). Larger datasets and/or more predictors are needed for better model development/validation and improved generalizability. These aspects are of great value as suboptimal clinical prediction models are responsible for research waste [57, 73]. Comparisons of SNNs with conventional regression models should be made in a fair manner, with the conventional models fully developed and interactions and/or nonlinear terms included when appropriate.

When comparing SNN methods to traditional approaches in simulation, scenarios with different sample sizes, censoring percentages, and numbers of covariates (fixed and/or time-varying) can be considered. Comparing SNNs in low and high dimensional settings is relevant to areas of study like bioinformatics. ANNs are often referred to as “black boxes”, due to the lack of interpretability (ANNs do not provide coefficients/hazard ratios as a Cox model does). The more complicated (deep) an ANN is, the more challenging interpretation of results becomes. As interpretability is necessary for clinical decision making, more emphasis should be placed on the development of methods which can facilitate SNN model interpretation. In section “sec:applications”, we discussed several applications that attempt to address this aspect. Olden et al.'s article provides a comparison of different techniques for ANN interpretability (e.g., variable importance) [74].

In the studies considered in this review, variability of performance (e.g., through the use of confidence intervals) was not well documented. The studies that did employ confidence intervals, typically used a resampling approach. Multiple resampling of all empirical data using bootstrapping can be an advantageous approach when sample size is limited, as it avoids the need to split the data for model development. While confidence intervals are necessary for model assessment, obtaining them can be computationally expensive. Further methods and guidelines for obtaining confidence intervals are needed. Another aspect which is under-reported in studies concerns the stability of SNN. ML techniques are algorithmic approaches that inherently rely on random processes to obtain generalisable models (e.g., for ANNs, values of weights are randomly initialized). Consequently, when rerunning the same model on the same data, there will be variations in output. In the event of a well-tuned model, these variations will be small and the model can be described as stable. In contrast, an incorrect approach to hyperparameter tuning may result in an unstable model with large variations. When validating an SNN, we recommend rerunning the model several times under the same parameterisation, to evaluate the stability of network's performance.

In section “sec:methodologies”, 13 methodologies were presented for survival prediction with SNNs. Some studies predict survival probabilities in the units of the output layer, which allows the estimated survival curves to be nonmonotonic (such networks cannot be forced to generate monotonically decreasing output units that predict survival probabilities) [10]. This can be avoided by predicting conditional hazard probabilities instead (from which survival probabilities can be readily calculated), as it is done, for example, in the PLANN and PLANN-ARD methods. We recommend that future ANN methodologies either estimate the (smoothed) hazard function in the output unit(s), or alternatively add constraints to ensure monotonicity of the survival curve. Furthermore, in this review, all neural networks were developed for right-censored data. Future work should focus on building SNNs for other types of censoring such as left or interval censoring, which are less common in practice compared to right censoring.

SNNs developed in recent years usually have more complicated structures and make use of multiple hidden layers (deep learners). It should be noted, however, that increasing the complexity of an ML prediction model does not necessarily translate to improved performance on new clinical data. An increase in the complexity, and by extent flexibility of a network may produce a model that is too attuned to the training data with poorer generalization to new data (overfitting), resulting in less accurate survival probabilities than a simpler network. Additionally, increasing complexity will pose additional challenges regarding interpretation. For clinical survival data using prognostic factors, sample size and number of predictors is likely to be insufficient for employing such advanced ML techniques. This may explain the frequent use of PLANNs in applications, as a PLANN guarantees survival curve monotonicity, relaxes proportional hazard assumption and employs a relatively simple network structure.

## 7. Conclusions

Nowadays, prediction models are ubiquitous in a wide range of research fields (e.g., medicine, engineering, and finance) and are becoming increasingly relevant in the medical field, as a result of the large-scale data collection and the increase in biological knowledge. In this paper, we discussed clinical prediction models with SNNs in the healthcare domain using prognostic factors, which can be used as guidance for future works. Light was shed on SNN approaches developed and applied from 1990 to August 2021. We assessed various methodological and practical aspects, including study characteristics, model development/validation, and comparison with Cox models. It is our opinion that, in the future, artificial intelligence and related algorithms (e.g., ANNs and SNNs) might become an integral part of personalised and evidence-based medicine. This review and critical appraisal hopely provides enough stimuli for researchers to be inspired from these methods and seek for new developments.

## Figures and Tables

**Table 1:** Notations used in this review.

Notation	Description
*T*	Survival time
*T*_max_	Maximum follow-up time (in years)
*q*_*k*_	Conditional survival probability in (output) unit *k*
*p*_*k*_	Conditional event probability in (output) unit *k*, with *p*_*k*_ = 1 − *q*_*k*_
*O*_*k*_	Output unit *k*
*w*	Connection weight matrix
*β*	Vector of regression coefficients
*x*	Covariate matrix
*x*_*i*_	Vector of *p* covariates for individual *i*
*Y*_*ki*_	Observed outcome of individual *i* in unit *k*
*ϕ*_*h*_	Activation function for the hidden layer
*ϕ*_*o*_	Activation function for the output layer
*α*	Bias unit (node)
*E*	Error (loss) function for the ANN
*δ*_*ik*_	Event indicator of individual *i* for time interval *k* = 1, ⋯, *K*
*p*_*ki*_	Probability that patient *i* relapses in time period (interval) *k*
*γ*_*k*_	Cumulative event probability in (output) unit *k*

**Table 2:** General characteristics for the 24 studies. If multiple outcomes were predicted, multiple lines were used in the extraction sheet. Maximum number of lines was 34 (10 studies used multiple outcomes). For simulation studies, the number of predictors and percentage of events were not considered, unless they were fixed (e.g., not varied across simulations).

	Min	1^*st*^ Qu.	Median	3^*rd*^ Qu.	Max	Excel lines
Total sample size	96	242	920	1616	361239	33
# of predictors	1	5	7	25.75	97	32
% of events	6.60	21.32	29.25	47.58	97.90	20

**Table 3:** The performance measures used for model validation across the 24 studies.

Performance criterion	N (%)
C-index	7 (29.2%)
AUC	5 (20.8%)
Log-likelihood	3 (12.5%)
Accuracy	2 (8.3%)
Global Chi-squared statistic of Cox regression	2 (8.3%)
Brier score	1 (4.2%)
Comparison of predicted probabilities with Kaplan-Meier	1 (4.2%)
Integrated brier score (IBS)	1 (4.2%)
Mean absolute error (MAE)	1 (4.2%)
McNemar's test	1 (4.2%)
Mean squared error (MSE)	1 (4.2%)
Prognostic risk group discrimination	1 (4.2%)
Sensitivity	1 (4.2%)
Separation of cases into good and bad prognosis	1 (4.2%)
Specificity	1 (4.2%)
Survival curves comparison with log-rank test	1 (4.2%)
Time-dependent C-index (*C*^*td*^)	1 (4.2%)
Wilcoxon test (separation of cases into good and bad prognosis)	1 (4.2%)

**Table 4:** Summary of the findings from the critical appraisal across the 24 manuscripts.

Unclear addressing of missing data (42.9%) or ad-hoc methods (23.8%)
Unclear reporting of hyperparameters (62.5%)
Unclear reporting of the performance criterion for model development (25.0%)
Unclear scaling of prognostic factors (41.7%)
Unclear programming language for SNNs (29.2%)
Large variability and improper performance measures for survival data
External validation for only 4 outcomes (11.8%)
No confidence intervals for the predictive measures (54.2%)
No calibration plots (54.2%)
No interactions in Cox regression or unclear reporting (89.5%)

## Data Availability

The excel sheets (SNNs review - short, SNNs review - long) developed for the critical appraisal of the 24 studies are provided in the online version of this manuscript.

## Abbreviations

ANN: Artificial neural network
AUC: Area under the curve
DNN: Deep neural network
FFANN: Feed forward artificial neural network
ML: Machine learning
PLANN: Partial logistic artificial neural network
PLANN-ARD: Partial logistic artificial neural network-automatic relevance determination
PLANNCR: Partial logistic artificial neural network for competing risks
PLANNCR-ARD: Partial logistic artificial neural network for competing risks-automatic relevance determination
RNN: Recurrent neural network
SNN: Survival neural network.
