# Supplementary material for: Neural Networks for Survival Prediction in Medicine Using Prognostic Factors: A Review and Critical Appraisal
Source: Comput Math Methods Med. 2022 Sep 30;2022:1176060. doi: 10.1155/2022/1176060 (PMC9553343; doi:10.1155/2022/1176060)
Supplement: Supplementary Materials — They are provided in the online version of this manuscript. [file 1176060.f1.zip › 1176060.f1.docx]

Neural networks for survival prediction in medicine using prognostic factors: a review and critical appraisal

Georgios Kantidakis, Audinga-Dea Hazewinkel, Marta Fiocco

SUPPLEMENTARY MATERIAL

Corresponding author:

Georgios Kantidakis

Leiden University Medical Center (LUMC)

[G.Kantidakis@lumc.nl](mailto:G.Kantidakis@lumc.nl)

# **Search string in PubMed**

”neural networks, computer” [MeSH Terms] AND ”survival analysis”[MeSH Terms] AND (prediction [All Fields] OR predictions[All Fields]) AND (”1990/01/01”[PDAT] : ”2021/08/31”[PDAT]). This string identified 261 potentially relevant studies based on our last search 01/09/2021.

# **Inclusion / exclusion criteria**

Studies were eligible if the article

• described the development and/or validation of a prediction model dealing with censored observations using prognostic factors in medical areas with Survival Neural Networks.

• described the application of an existing methodology of Survival Neural Networks for survival data using prognostic factors in real-world medical data or simulation studies.

Of particular interest is the comparison of the predictive performance with the Cox models which are the benchmark for survival analysis.

Studies were excluded if

• the models were developed for non-humans

• the predictions were based on individual’s images (pathology images, tumor slices, whole slide images, MRI etc.)

• the ML techniques / the ANNs were built for standard classification or regression

• there was bioinformatics / computational biology analysis (predictions for gene signatures or gene expression profiling)

• the models focused on feature selection / reduction

• the models were developed using unsupervised ML techniques (e.g. data mining, text mining)

• the models were built using other ML techniques for survival analysis (e.g. Random Survival Forest)

• ML techniques were used for risk group stratification only

• the predictions were built based on an ensemble of different ML techniques (pipeline)

• there was no prediction (no evaluation of model’s performance)

• the models replaced the linear function of a Cox model by the output of a neural network (extensions of Cox regression)

• the models focused on a new prediction tool (e.g. nomograms, partin tables)

• ANNs were built only for the distribution of the survival times or the martingale residuals

• ANNs were built based on a form of response transformation

• ANNs were built for dynamic survival analysis (joint-modelling of longitudinal and time-to-event data)

• they presented a systematic / literature review in medicine

• they presented a tutorial / case study

• they presented a letter to the editor

# **Extracted items for each study**

| **Item** | **Comment** |
| --- | --- |
| Title | The title of the study |
| DOI | Digital object identifier |
| Manuscript type | Classification of the manuscript as methodological or application |
| Author | The first author of the publication |
| Journal | Name of the journal in which the study was published |
| Publication year | The year of the publication of the study in that journal |
| Medical field | The field of the real-world data or the simulation study |
| # patients | Number of patients for a particular dataset |
| # predictors | Number of predictors for a particular dataset |
| # outcomes | Number of endpoints analyzed, and which endpoints |
| Events % | Percentage of events in a particular dataset |
| Max follow-up time | The maximum of the follow-up time for the study in a particular dataset |
| How were missing values (if any) addressed in the prognostic factors? | The method applied to deal with missing values in the study. This is not applicable for the simulation studies. |
| How were prognostic variables scaled for NN? | Approach for scaling of the prognostic factors or no scaling needed otherwise |
| Hyperparameters | How were the hyperparameters addressed for the neural networks (tuned or default values)? Was the procedure followed unclear? |
| Performance criterion (model development)? | The performance criterion for the NNs (criterion applied to train the model) |
| Performance criterion (model validation)? | The performance criterion for the NNs (criterion applied to validate the performance of the model) |
| Method for confidence intervals for NN? | Which was the method that the authors followed to construct confidence intervals, or no confidence interval otherwise |
| Validation | Which was the validation procedure in the study? (e.g. split sample approach, resampling, external validation) |
| Calibration plot | Did the authors assess calibration with a calibration curve? |
| Programming language used for NN | Which was the programming language used to fit the neural network(s) |
| Claim for NN performance compared to Cox model? | What did the authors claim regarding the performance of the neural network compared to the conventional Cox model? (if applicable) |
| Interactions in Cox regression? | Were any interaction terms included for the Cox regression? (if applicable) |

Table A. Overview of extracted items for each study. When an item was not reported or it could not be identified by the first author, it was specified as “unclear”. Initially, an excel sheet was created with the detailed extracted items. Afterwards, we created supplementary excel sheets uniformizing the levels of the extracted items as presented in the next pages of this supplementary material.

# **Study characteristics**

| **Classification of the manuscripts** | **N (%)** |
| --- | --- |
| Methodology | 13 (54.2%) |
| Application | 11 (45.8%) |
| **How were the prognostic factors scaled?** |  |
| Unclear | 10 (41.7%) |
| No scaling needed | 7 (29.2%) |
| Normalization | 5 (20.8%) |
| Standardization | 2 (8.3%) |
| **Confidence intervals (CIs)** |  |
| No CI | 13 (54.2%) |
| Data resampling 10 times | 2 (8.3%) |
| Data resampling 100 times | 2 (8.3%) |
| Data resampling 50 times | 2 (8.3%) |
| Unclear | 2 (8.3%) |
| A non-parametric CI based on Gaussian approximation was obtained by jack-knifing the empirical estimator Ctd | 1 (4.2%) |
| Repeat simulations 500 times | 1 (4.2%) |
| Rerun neural network 10 times for each covariate | 1 (4.2%) |
| **Calibration plots** |  |
| No | 13 (54.2%) |
| Yes | 11 (45.9%) |

Table A.1. Manuscript grouping, prognostic factor scaling, how the confidence intervals were constructed, and whether a calibration plot was created. (N = 24 studies).

| **Year of publication** | **N (%)** |
| --- | --- |
| 1992 | 1 (4.2%) |
| 1994 | 2 (8.3%) |
| 1995 | 1 (4.2%) |
| 1998 | 2 (8.3%) |
| 2000 | 1 (4.2%) |
| 2003 | 2 (8.3%) |
| 2004 | 1 (4.2%) |
| 2006 | 1 (4.2%) |
| 2007 | 2 (8.3%) |
| 2008 | 2 (8.3%) |
| 2013 | 2 (8.3%) |
| 2018 | 3 (12.5%) |
| 2019 | 2 (8.3%) |
| 2020 | 2 (8.3%) |

Table A.2. Year of publication for the 24 selected studies.

| **Performance criterion (model development)** | **N (%)** |
| --- | --- |
| log-likelihood | 5 (20.8%) |
| C-index | 3 (12.5%) |
| AUC | 2 (8.3%) |
| Bayesian Information Criterion (BIC) | 1 (4.2%) |
| Cross-entropy | 1 (4.2%) |
| Global Chi-squared statistic of Cox regression | 1 (4.2%) |
| Integrated Brier Score (IBS) | 1 (4.2%) |
| Mean Absolute Error (MAE) | 1 (4.2%) |
| McNemar's test | 1 (4.2%) |
| Network Information Criterion (NIC) | 1 (4.2%) |
| Relative entropy | 1 (4.2%) |
| Root Mean Squared Error (RMSE) | 1 (4.2%) |

Table A.3. The performance criteria used for model development across the 24 studies. The training criterion was unclear for 6 studies (25.0%).

| **Performance criterion (model validation)** | **N (%)** |
| --- | --- |
| C-index | 7 (29.2%) |
| AUC | 5 (20.8%) |
| log-likelihood | 3 (12.5%) |
| Accuracy | 2 (8.3%) |
| Global Chi-squared statistic of Cox regression | 2 (8.3%) |
| Brier Score | 1 (4.2%) |
| Comparison of predicted probabilities with Kaplan Meier | 1 (4.2%) |
| Integrated Brier Score (IBS) | 1 (4.2%) |
| Mean Absolute Error (MAE) | 1 (4.2%) |
| McNemar's test | 1 (4.2%) |
| Mean Squared Error (MSE) | 1 (4.2%) |
| Prognostic risk group discrimination | 1 (4.2%) |
| Sensitivity | 1 (4.2%) |
| Separation of cases into good and bad prognosis | 1 (4.2%) |
| Specificity | 1 (4.2%) |
| Survival curves comparison with log-rank test | 1 (4.2%) |
| Time dependent C-index (C^td^) | 1 (4.2%) |
| Wilcoxon test (separation of cases into good and bad prognosis) | 1 (4.2%) |

Table A.4. The performance measures used for model validation across the 24 studies.

| **How were missing values (if any) addressed for the 21 studies?** | **N (%)** |
| --- | --- |
| Unclear | 9 (42.9%) |
| k-nearest neighbor | 2 (9.5%) |
| Separate attribute | 2 (9.5%) |
| missForest algorithm | 1 (4.8%) |
| Multiple imputations by fully conditional specification | 1 (4.8%) |
| Multiple regression | 1 (4.8%) |
| No missing values | 1 (4.8%) |
| Probability imputation 20 times | 1 (4.8%) |
| Separate attribute for categorical, ignored for continuous | 1 (4.8%) |
| Separate attribute for categorical, mean imputation for continuous | 1 (4.8%) |
| Separate attribute for categorical, median imputation for continuous | 1 (4.8%) |

Table A.5. Approaches to deal with potential missing data for the 21 existing data studies. 3 studies were not taken into account regarding simulated datasets.

| **Programming language for ANN** | **N (%)** |
| --- | --- |
| Unclear | 7 (29.2%) |
| Python | 4 (16.7%) |
| Matlab | 3 (12.5%) |
| NeuralWare | 3 (12.5%) |
| S-plus | 3 (12.5%) |
| R | 2 (8.3%) |
| Epilog Plus | 1 (4.2%) |
| PlaNet | 1 (4.2%) |
| **Hyperparameters** |  |
| Tuned – unclear procedure | 7 (29.2%) |
| Unclear | 6 (25.0%) |
| Tuned | 4 (16.7%) |
| Default and tuned | 3 (12.5%) |
| Default | 2 (8.3%) |
| Default and tuned – unclear procedure | 2 (8.3%) |

Table A.6. The programming language used and hyperparameters for the ANN (N = 24 studies).

| **Claim for the performance of ANN compared to Cox model (19 studies with comparison)** | **N (%)** |
| --- | --- |
| Better | 9 (47.4%) |
| Same or better | 5 (26.3%) |
| Not better | 3 (15.8%) |
| Same | 2 (10.5%) |
| **Interactions in Cox regression** |  |
| No | 15 (78.9%) |
| Unclear | 2 (10.5%) |
| Yes | 2 (10.5%) |

Table A.7. Comparison for the performance of the ANN with Cox model according to the authors and whether interactions were tested in Cox regression. (N = 19 studies)

--------------------------------------------------------------------------------------------------------------------------------------

Statistics for the 34 lines of the excel sheet in long format. If multiple outcomes are predicted, multiple rows are used (real world or simulated datasets). Ten studies are used more than once.

|  | **Min.** | **1^st^ Qu.** | **Median** | **3^rd^ Qu** | **Max** | **Excel rows^*^** |
| --- | --- | --- | --- | --- | --- | --- |
| **Total sample size ^a^** | 96 | 242 | 920 | 1616 | 361239 | 33 |
| **Number of predictors ^b^** | 1 | 5 | 7 | 25.75 | 97 | 32 |
| **% of events ^c^** | 6.60 | 21.32 | 29.25 | 47.58 | 97.90 | 20 |

**^*^** ^The maximum number of rows is 34 (10 studies used more than once). Cells of simulation studies where the number of predictors / the percentage of events is not fixed are not included.^

**^a^** ^Number using the full dataset.^

**^b^** ^In some cases the number of predictors was not mentioned explicitly but it could reasonably be derived.^

**^c^** ^10 cells with unclear % of events are not included in the descriptive statistics.^

Table A.8. Descriptive statistics for the total sample size, the number of predictors, and the percentage of events.

| **Medical field** | **N (%)** |
| --- | --- |
| Breast cancer | 10 (29.4%) |
| General (simulation study) | 3 (8.8%) |
| Cardiovascular disease | 2 (5.9%) |
| Cervical cancer | 2 (5.9%) |
| Gastric cancer | 2 (5.9%) |
| Lung cancer | 2 (5.9%) |
| Prostate cancer | 2 (5.9%) |
| Colorectal cancer | 1 (2.9%) |
| Coronary artery disease | 1 (2.9%) |
| Head and neck cancer | 1 (2.9%) |
| Laryngeal squamous carcinoma | 1 (2.9%) |
| Liver transplantation | 1 (2.9%) |
| Malignant melanoma | 1 (2.9%) |
| Pharmacometrics (simulation study) | 1 (2.9%) |
| Post-partum amenorrhea | 1 (2.9%) |
| Renal cell carcinoma | 1 (2.9%) |
| Synovial sarcoma | 1 (2.9%) |
| Uveal melanoma | 1 (2.9%) |
| **Study outcomes** |  |
| Overall survival | 16 (47.1%) |
| Recurrence-free survival | 5 (14.7%) |
| Disease-free survival | 3 (8.8%) |
| Relapse-free survival | 3 (8.8%) |
| Breast cancer specific mortality | 2 (5.9%) |
| Death or hospitalization due to cardiovascular events | 2 (5.9%) |
| Menstruation-free survival after birth | 1 (2.9%) |
| Progression-free survival | 1 (2.9%) |
| Time to clinical artery events | 1 (2.9%) |
| **Validation approach** |  |
| Single random split | 17 (50.0%) |
| Resampling – cross validation | 7 (20.6%) |
| External validation | 4 (11.8%) |
| Multiple random split | 2 (5.9%) |
| No validation | 2 (5.9%) |
| Resampling – nested cross validation | 2 (5.9%) |

Table A.9. Medical field, study outcomes and validation approach for 34 excel lines in long format for the 24 studies.
